# SARS-CoV-2 Infection Alters the Phenotype and Gene Expression of Adipocytes

**DOI:** 10.3390/ijms25042086

**Published:** 2024-02-08

**Authors:** Paola Quaranta, Gaia Scabia, Barbara Storti, Alessia Dattilo, Lara Quintino, Paola Perrera, Cristina Di Primio, Mario Costa, Mauro Pistello, Ranieri Bizzarri, Margherita Maffei

**Affiliations:** 1 Retrovirus Center, Virology Section, Department of Translational Research and New Technologies in Medicine and Surgery, University of Pisa, Via Savi 10, 56126 Pisa, Italy; paola.quaranta@unipi.it (P.Q.); paola.perrera@gmail.com (P.P.); mauro.pistello@unipi.it (M.P.); 2 National Research Council—Institute of Neuroscience, Via Moruzzi 1, 56124 Pisa, Italy; cristina.diprimio@cnr.it (C.D.P.); costa@in.cnr.it (M.C.); 3 National Research Council—Institute of Clinical Physiology, Via Moruzzi 1, 56124 Pisa, Italy; gaia.scabia@cnr.it (G.S.); quintinolara96@gmail.com (L.Q.); 4 Center for Obesity and Lipodystrophy, Pisa University-Hospital, Via Paradisa 2, 56124 Pisa, Italy; lelle896@gmail.com; 5 National Enterprise for nanoScience and nanoTechnology, Scuola Normale Superiore, National Research Council—Institute of Nanoscience, Piazza San Silvestro 12, 56127 Pisa, Italy; ranieri.bizzarri@unipi.it; 6 Virology Unit, Pisa University-Hospital, Via Paradisa 2, 56124 Pisa, Italy; 7 Department of Surgical, Medical and Molecular Pathology, and Critical Care Medicine, University of Pisa, Via Roma 65, 56126 Pisa, Italy; 8 Italian National Institute for Nuclear Physics, Via Filippo Buonarroti 3, 56127 Pisa, Italy

**Keywords:** COVID-19, lipid droplet, SGBS, inflammation

## Abstract

Epidemiological evidence emphasizes that excess fat mass is associated with an increased risk of severe COVID-19 disease. Nevertheless, the intricate interplay between SARS-CoV-2 and adipocytes remains poorly understood. It is crucial to decipher the progression of COVID-19 both in the acute phase and on long-term outcomes. In this study, an in vitro model using the human SGBS cell line (Simpson-Golabi-Behmel syndrome) was developed to investigate the infectivity of SARS-CoV-2 in adipocytes, and the effects of virus exposure on adipocyte function. Our results show that SGBS adipocytes expressing ACE2 are susceptible to SARS-CoV-2 infection, as evidenced by the release of the viral genome into the medium, detection of the nucleocapsid in cell lysates, and positive immunostaining for the spike protein. Infected adipocytes show remarkable changes compared to uninfected controls: increased surface area of lipid droplets, upregulated expression of genes of inflammation (*Haptoglobin*, *MCP-1*, *IL-6*, *PAI-1*), increased oxidative stress (MnSOD), and a concomitant reduction of transcripts related to adipocyte function (leptin, fatty acid synthase, perilipin). Moreover, exogenous expression of spike protein in SGBS adipocytes also led to an increase in lipid droplet size. In conclusion using the human SGBS cell line, we detected SARS-CoV-2 infectivity in adipocytes, revealing substantial morphological and functional changes in infected cells.

## 1. Introduction

The pathogen SARS-CoV-2, a member of the coronavirus family, is the causative agent behind the worldwide COVID-19 pandemic. This disease is characterized by a spectrum of clinical manifestations, ranging from asymptomatic infection to severe forms, for which extreme inflammation and cytokine storm have been implicated as potential contributors to fatal outcomes [1]. Notably, obesity, a condition often regarded as a global pandemic closely linked to poverty and socioeconomic disadvantage, emerges as an independent risk factor for severe and lethal forms of COVID-19 [2,3,4]. While patients with obesity commonly exhibit a spectrum of other conditions typical of the metabolic syndrome and are predisposed to an elevated risk of cardiovascular disease [5], it is noteworthy that comorbidities linked to excess weight, such as hypertension and type 2 diabetes, confer an independent relative risk for severe forms of COVID-19, albeit lower than that associated with obesity [3,6,7]. The excessive accumulation of fat mass could potentially serve as a trigger for the severe progression of COVID-19 itself. Notably, during obesity, fat mass undergoes substantial rearrangement, leading to a radical shift in the expression profile of adipocytes [8]. This alteration results in the overproduction of chemoattractant factors that recruit immune cells from the circulation, eliciting a state of low-grade chronic systemic inflammation [9]. This inflammatory condition primes the onset of the comorbidities associated with obesity. The critical entry point for the virus, the membrane-bound glycoprotein angiotensin-converting enzyme 2 (ACE2), is expressed in white adipose tissue (WAT). WAT has been implicated as a target for SARS-CoV-2 and a potential reservoir for prolonged viral shedding, suggesting a potential contribution to the outcome of “long COVID”. Notably, SARS-CoV-2 has been detected in postmortem specimens of white adipose tissue from infected subjects [10,11,12].

The connection between COVID-19 and obesity extends beyond the virus’ ability to enter adipocytes. Notably, embolic fat has been identified in the lungs of individuals with obesity who succumbed to COVID-19 [13]. The authors posit that a substantial source of these lipids may originate from infected, dying adipocytes. This observation raises critical implications for understanding the effects of adipose tissue infection on the response to COVID-19 in distant regions, such as the respiratory apparatus.

A deeper comprehension of the interaction between the coronavirus and the adipocyte is pivotal for addressing the evolution of COVID-19, both in its acute phase and considering the increasingly recognized long-term effects that have become a critical issue to tackle.

Adipocytes are primarily characterized by the presence of lipid droplets (LD), vacuoles filled with heterogeneous triglycerides enclosed by a phospholipid monolayer and adorned with a specific set of proteins, with perilipin 1 (PLIN1) being the most abundant. While in vivo adipocytes typically feature a single lipid vacuole, cultured adipocytes never reach this condition, and instead exhibit the presence of multiple LDs. SARS-CoV-2 exhibits a heightened capacity for mediating cell–cell fusion, a notable distinction from the previously emerged SARS-CoV [14]. This fusion is facilitated by the Spike (S) protein, a crucial target for antiviral interventions responsible for viral receptor binding and membrane fusion. Notably, the classification of SARS-CoV-2 variants also takes into account their membrane fusion kinetics, indicating potential differences in pathogenicity [15].

Taking the aforementioned into account, it is plausible that the increased susceptibility to adverse outcomes in COVID-19 among individuals with obesity results from a complex interplay between the virus and alterations in adipocytes.

This study aims to explore the suitability of the preadipocyte/adipocyte human cell line SGBS for investigating the effects of SARS-CoV-2 on the adipose organ. Our assessment will focus on morphological changes and alterations in gene expression.

## 2. Results

### 2.1. SGBS Cells Are Permissive to SARS-CoV-2 Infection

**Figure 1:**
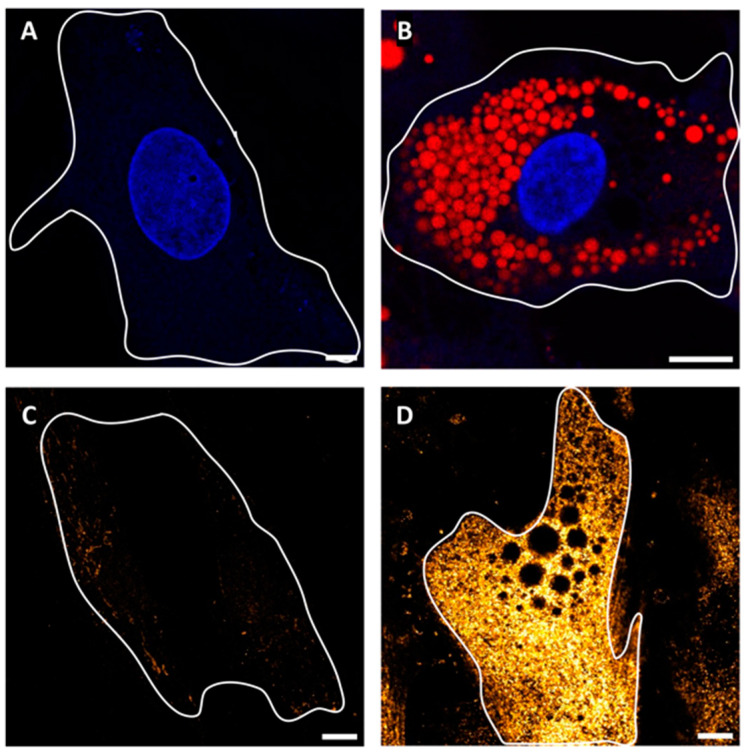
Phenotypes of SGBS pre-adipocytes and adipocytes, as revealed by confocal microscopy. (**A**,**B**) One SGBS pre-adipocyte (**A**) and one SGBS adipocyte (**B**) stained by Hoechst and Oil red, and confocally imaged at the medial plane by collecting fluorescence in the blue and far-red channels. (**C**,**D**) One SGBS pre-adipocyte (**C**) and one SGBS adipocyte (**D**) immunostained for ACE2 (primary Ab: rabbit antiACE2, secondary Ab: αr488) confocally imaged at the membrane plane by collecting fluorescence in the green channel. Fluorescence intensity was coded by a pseudo-color FireHot scale to pinpoint the strong difference in expression levels between the two cell types. In all cases, the cell’s contours were highlighted by a white line. Scale bar: 10 μm.

**Figure 2:**
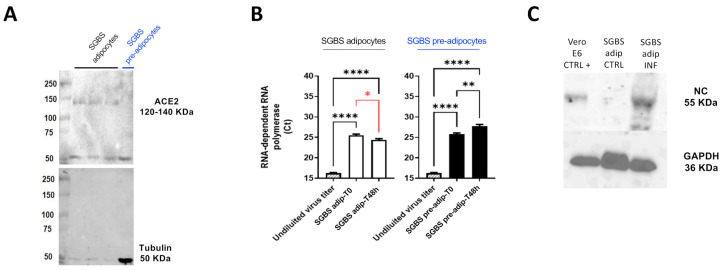
Differentiated SGBS cells are permissive for SARS-CoV-2 infection. (**A**) ACE2 protein expression in SGBS adipocytes (black label) and pre-adipocytes (blue label); (**B**) RNA-dependent RNA polymerase Ct in SGBS adipocytes (left, white bars) and pre-adipocytes (right, black bars), with respect to undiluted virus titer (striped bars). Cts are significantly decreased after 48 h in PID10 cells (SGBS adip) in respect to T0 (red asterisk); (**C**) Nucleocapsid protein expression in infected VERO E6 cells (positive control, left lane) and not infected (negative control, middle lane) and infected (right lane) SGBS adipocytes. One-way analysis of variance followed by Dunnett’s multiple comparison test: **** *p* < 0.0001; ** *p* < 0.01; * *p* < 0.05.

SGBS cells are a non-immortalized cell strain originally isolated from the subcutaneous WAT of a patient with Simpson–Golabi–Behmel syndrome and reflecting key features of human primary adipose-derived stromal cells [16,17]. In the undifferentiated stage (post-induction day 0, from now on referred to as PID0 or pre-adipocytes) they appear as normal fibroblasts and, when induced with an adipogenic cocktail, undergo changes in gene expression and cellular morphology that lead to the accumulation of triglycerides in the form of multiple lipid droplets (from now on referred to as adipocytes or PID10). Of note, staining of adipocytes with Oil Red O enables their efficient imaging by a confocal microscope by collecting far-red fluorescence emissions (Figure 1A,B). Given that their response to SARS-CoV-2 infection was never reported, we wanted to investigate if they were permissive to the virus infection. Our initial focus was on tracking the expression of ACE2, the main receptor for virus entry into the cell. *ACE2* mRNA was weakly detectable in both adipocytes and preadipocytes. However, as illustrated in Figure 2A, while the ACE2 protein is not expressed in the latter, its expression is detectable in the former. This finding was confirmed by the confocal microscopy of cells immunostained for ACE2, which showed a large difference between the fluorescence signals of SGBS adipocytes and pre-adipocytes (Figure 1C,D).

Subsequently, we employed the Wuhan strain (B.1) of the coronavirus to infect SGBS cells at PID0 and at PID10, and measured the release of viral particles into the supernatant as a key indicator of productive infection. In Figure 2B, we note a distinctive pattern in the real-time cycle thresholds (Ct) that are inversely related to the abundance of SARS-CoV-2 mRNA. Notably, 24 h post-infection, there is a discernible drop in Cts in differentiated SGBS, but not in undifferentiated counterparts. This observation suggests that only the differentiated SGBS cells are actively engaged in the production of the virus, distinguishing them from the undifferentiated cells in this regard.

To gain further additional independent evidence in this direction, we conducted a Western blot analysis to examine the presence of the viral nucleocapsid protein (NC), which was consistently detected in infected adipocytes and in VERO E6 cells, the latter serving as a positive control [18,19] (Figure 2C).

**Figure 3:**
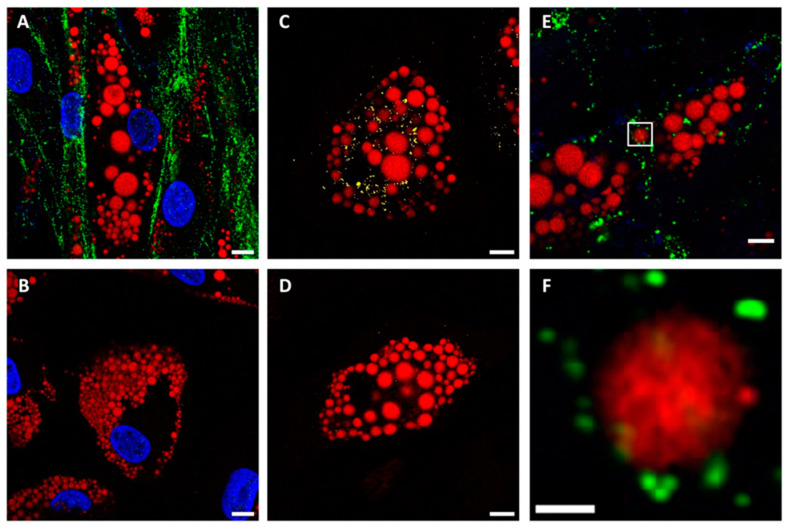
SARS-CoV-2 infection of SGBS pre-adipocytes and adipocytes, as revealed by confocal and super-resolution microscopy. (**A**–**D**) Confocal imaging of SGBS adipocytes exposed (**A**,**C**) and not exposed (**B**,**D**) to SARS-CoV-2 and immunostained for the S protein ((**A**,**B**), green) and the N protein ((**C**,**D**), yellow). Red signal: lipid droplets (Oil red); blue signal: Hoechst. (**E**,**F**) Super-resolution (Image Scanning Microscopy/Airyscan) images of infected adipocytes (green: Spike protein, red: lipid droplets). The portion of the image surrounded by the white square in (**E**) is magnified in (**F**). Scale bar: 10 μm.

Positive infection of adipocytes was confirmed by confocal imaging: both Spike (Figure 3A, green color) and NC proteins (Figure 3C, yellow color) were detected in infected cells, as compared to control cells not exposed to the virus (Figure 3B,D). Super resolution imaging at mesoscale (100–140 nm) [20] by Image Scanning Microscopy (Airyscan mode) enabled the visualization of single viral particles [21], some of which were found in extremely close proximity to the lipid droplets (LDs) (Figure 3E,F, red color).

Collectively, these findings establish that differentiated SGBS adipocytes are susceptible to SARS-CoV-2 infection, underscoring their potential utility as a model for investigating the virus’s impact on the adipose organ.

### 2.2. The Effects of SARS-CoV-2 Infection on SGBS Morphology and Gene Expression

**Figure 4:**
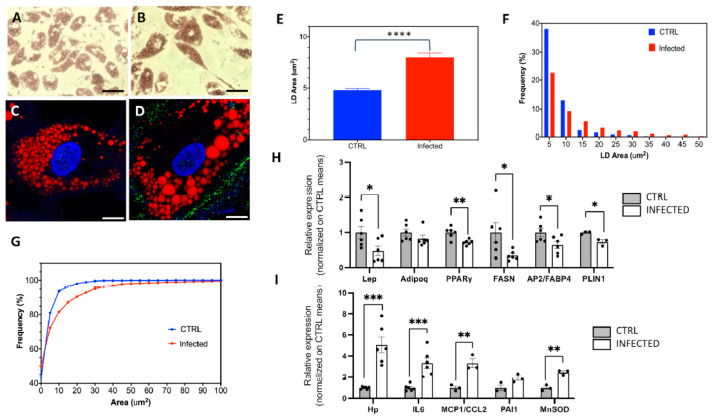
SARS-CoV-2 infection of SGBS adipocytes induces enlargement of LDs, as revealed by confocal microscopy. (**A**,**B**) Transmitted light microscopy images of SGBS adipocytes not exposed (**A**) and exposed (**B**) to SARS-CoV-2 stained with Oil red O (20× magnification; scale bar: 50 μm). (**C**,**D**): Confocal imaging of SGBS adipocytes exposed (**C**) and not exposed (**D**) to SARS-CoV-2 and immunostained for the S protein (green); red signal: lipid droplets (Oil red); blue signal: Hoechst; scale bar: 10 μm. (**E**) Column bar graph showing mean and SEM of LD areas in CTRL and infected cells (CTRL: 4.8 ± 0.2 μm^2^, #1182 LDs; infected: 8.1 ± 0.4 μm^2^. #1325 LDs); ****: *p* < 0.0001 (*t*-test). (**F**,**G**) Frequency distributions (**F**) and cumulative distributions (**G**) of LD areas in CTRL and infected cells; the cumulative distributions were statistically different (*p* < 0.0001), as assessed by the Kolmogorov–Smirnov test. (**H**,**I**) CTRL and infected SGBS adipocytes’ expression of terminal adipocyte differentiation markers (**H**) (*Lep*, *Adipoq*, *PPARγ*, *FASN*, *ap2/FABP4* and *PLIN1* and inflammatory and oxidative stress markers (**I**) (*Hp*, *IL-6*, *MCP1/CCL2*, *PAI-1* and *MnSOD*). Student’s *t*-test: *** *p* < 0.001; ** *p* > 0.01; * *p* < 0.5.

Next, we focused on differentiated SGBS only, and examined the influence of SARS-CoV-2 on the adipocyte phenotype and gene expression. The microphotographs captured at both low and high magnifications using light transmission and fluorescence, respectively, (Figure 4A–D) revealed a notable enlargement of LDs in infected cells as compared to controls. To verify this effect, we carried out a morphometric assessment on confocal images involving the intensity-based segmentation of single LDs. Analysis of over 1000 LDs indicated that exposure to SARS-CoV-2 leads to the significant enlargement of the LDs, with a fairly higher dispersion in infected cells (Figure 4E). Comparison of the area distributions clearly confirmed the statistically significant higher representation of larger LDs in the presence of infection (Figure 4F,G). Such enlargement may occur in cells that heighten their polarization toward adipogenesis, typically correlated with an increased abundance of transcripts marking the terminal differentiation of the adipocyte. These elements encompass the transcription factor *PPARγ*, the adipokines *leptin* and *adiponectin*, the crucial enzyme for triglyceride biosynthesis *fatty acid synthase* (*FASN*), the lipid transport protein *FABP4*, and the protein involved in LD homeostasis *Perilipin 1* (*PLIN1*). However, droplet digital PCR (ddPCR) analysis of the abundance of these mRNAs revealed no increase; on the contrary, a significant decrease was observed in SARS-CoV-2-infected adipocytes for *PPARgamma*, *FASN*, *leptin*, *FABP4*, and *PLIN1*. This excludes their involvement in the observed enlargement of lipid vacuoles (Figure 4H).

Subsequently, we aimed to monitor the expression of genes typically upregulated in inflamed/dysfunctional WAT, a condition known to increase the risk of developing insulin resistance and other metabolic alterations. These genes include the macrophage chemotactic factors *haptoglobin* and *MCP1*/*CCL2*, the cytokine *IL-6*, and *PAI-1*. Intriguingly, all of them exhibited a higher transcript abundance in infected cells. Additionally, we found an increased expression of *MnSOD*, the primary superoxide-scavenging enzyme that protects cells from the consequences of oxidative stress damage (Figure 4I).

The data suggest that SARS-CoV-2 infection has a remarkable impact on SGBS adipocyte expression, leading to the induction of an inflammatory profile and the repression of adipokine genes, as well as genes associated with triglyceride turnover.

### 2.3. The Role of Spike Protein in LD Enlargement

Having ruled out the virus-dependent induction of adipogenesis and/or triglyceride uptake/synthesis, we posited a hypothesis that the enlargement of LDs could be linked to the fusion of multiple organelles mediated by the viral spike protein, which, as mentioned above, has an established role in cell membrane fusion leading to the formation of pneumocytes and neuron syncytia [22,23,24].

**Figure 5:**
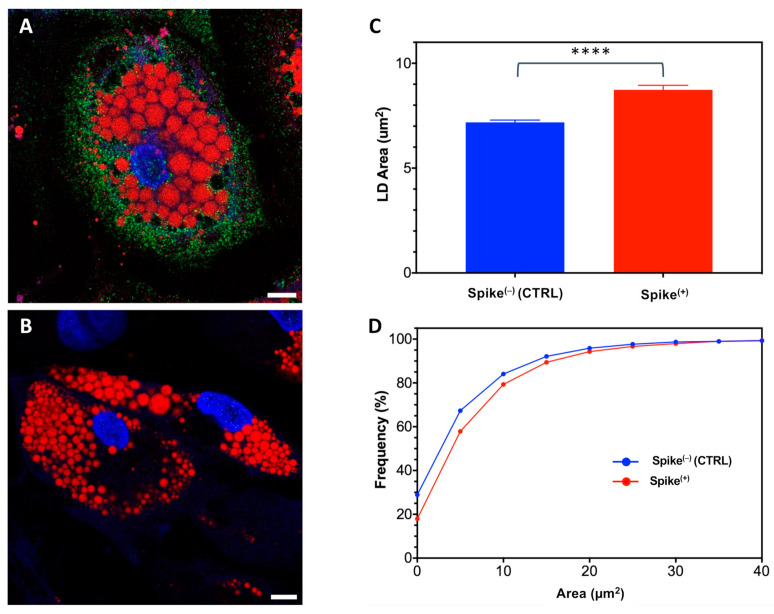
Exogenous expression of Spike protein in SGBS adipocytes induces enlargement of LDs as revealed by confocal microscopy. (**A**,**B**) Immunostaining followed by confocal imaging of SGBS adipocytes positive (Spike^(+)^, (**A**)) or negative (Spike^(−)^, (**B**)) for Spike protein (green). Red signal: lipid droplets (Oil red); blue signal: nuclei (Hoechst). Scale bar: 10 μm. (**C**) Column bar graph showing mean and SEM of LD areas in Spike^(−)^ and Spike^(+)^ cells (CTRL: 7.2 ± 0.1 μm^2^, #10106 LDs; infected: 8.7 ± 0.2 μm^2^, #7951 LDs. ****: *p* < 0.0001 (*t*-test). (**D**) Frequency cumulative distribution of LD areas in Spike^(−)^ and Spike^(+)^ cells; the cumulative distributions resulted statistically different (*p* < 0.0001) as assessed by the Kolmogorov-Smirnov test.

To explore this possibility, we conducted transient transfections of SGBS cells during their adipogenic conversion (PID3) using a plasmid encoding the spike cDNA. Subsequently, we allowed the Spike(+) cells to progress until terminal differentiation (PID10). Following the verification of Spike protein expression through immunostaining, we conducted an LD morphometric analysis (Figure 5).

Remarkably, larger LDs were found to be associated with the presence of the Spike protein, with high statistical significance. Of note, transient transfection with EGFP did not induce LD enlargement (Supplementary Figure S1), suggesting that the increase in size of LDs cannot be attributed to the non-specific transfection of the cells. Collectively, these findings provide support for our hypothesis of a specific fusogenic effect of Spike proteins on LDs.

## 3. Discussion

Our study demonstrates that differentiated human SGBS cells effectively support SARS-CoV-2 infection and serve as a platform for virus replication. In contrast, undifferentiated SGBS cells show no permissiveness to active virus infection. These findings are in agreement with Martínez-Colón and colleagues [12], who observed viral particles in both adipocytes and stromal vascular cells (SVC) isolated from WAT biopsies. However, only adipocytes, not SVC, exhibited permissiveness for viral replication. The notable presence of the ACE2 protein, a crucial entry point for viral infection, identified by us in differentiated SGBS cells, but not in undifferentiated ones, may partially explain the observed outcomes. Our results diverge somewhat from previous reports which indicate low or negligible ACE2 levels in both pre-adipocytes and differentiated adipocytes [12,13] and contextually suggest alternative entry factors for SARS-CoV-2 infection, including CD147, dipeptidyl peptidase 4, neuropilin 1 (NRP1), and FURIN [25,26]. The discrepancy in ACE2 expression between our study and others can be attributed to two key factors. Firstly, the use of different preadipocyte/adipocyte models, specifically human multipotent adipocyte stem cells (hMADS) or stromal vascular cells (SVC) versus SGBS cells. Secondly, the mentioned studies reported low *ACE2* transcripts, consistent with our findings, while we were able to detect the ACE2 protein, a facet not explored in those studies. An aspect worth mentioning is the occurrence of virus RNA packaging within double-membrane vesicles originating from the endoplasmic reticulum [27,28]. Intriguingly, LDs also emerge from the same cellular compartment [29]. This shared origin prompts speculation about the potential influence of these vesicles or the underlying mechanisms of their formation, suggesting a possibility that they may favor virus packaging and replication specifically within adipocytes. By harnessing the capabilities of the SGBS human cell line, our research has unveiled intriguing insights into the influence of SARS-CoV-2 on adipocyte function. These revelations encompass not only morphostructural changes, impacting the size of LDs—the main reservoirs of triglycerides—but also transcriptional alterations affecting genes linked to metabolism and inflammation. Notably, the induction of inflammatory genes stands out as a highly anticipated outcome, aligning with findings documented in prior literature about the induction of an inflammatory response in SARS-CoV-2-infected WAT. An elevated infiltration of inflammatory cells has been documented in post-mortem samples of WAT from COVID-19 patients [11,13]. The heightened expression of *MCP1* and *Haptoglobin* in infected adipocytes, as observed in our study, could potentially play a pivotal role in driving this infiltration. Previous studies [9,30,31] highlight, in fact, their significance as chemoattractant proteins for leukocytes and macrophages, suggesting a plausible mechanism for the observed higher inflammatory cell infiltration in COVID-19 patients’ WAT [11]. Similarly, the infection-induced increase of *PAI-1* may also contribute to macrophage infiltration [32]. In addition, elevated *PAI-1* levels have been linked to the impairment of insulin signaling in obesity and in the establishment of a chronic inflammatory state [33], similar to the role played by *IL-6*, also upregulated in our model. The data presented paint a picture of the response of adipocytes to a viral insult. On the one hand, it triggers the activation of the immune system and contributes to an increased systemic inflammatory status, while at the same time it strengthens its defenses against increased oxidative stress, as shown by the induction of *MnSOD* (superoxide dismutase) [34]. The expression profile of SGBS adipocytes undergoes significant alterations post-infection, manifesting in noteworthy changes, such as a pronounced downregulation of *leptin* and genes associated with lipid synthesis, including *PPARgamma*, *FASN*, *FABP4*, and *PLIN1.* PPARgamma, a pivotal transcription factor crucial for adipogenesis, plays a key role in triglyceride accumulation within adipocytes by initiating the lipogenesis machinery [35,36]. Consequently, the diminished expression of *PPARgamma* observed during SARS-CoV-2 infection appears to be a potential regulatory factor upstream of the observed decrease in the abundance of the other mentioned genes. These findings collectively suggest an overall impairment in adipocyte function induced by SARS-CoV-2 infection. Regarding leptin, it is worth mentioning that the “satiety hormone” has previously been reported to be elevated in both plasma and adipose tissue of COVID-19 patients [37,38]. The published analyses within the field employ a predominantly cross-sectional approach, neglecting the established and direct correlation between leptin and adipose mass, or leptin and increased risk of cardiovascular diseases (as extensively documented) [39,40]. Notably, adipose mass emerges as a determinant for the severe prognosis of COVID-19 [41], suggesting that the association with leptin may manifest as a secondary rather than a direct relationship. This observation holds significance, as leptin is recognized for its role in shaping Th cytokine production toward a proinflammatory phenotype (Th1, IFN) [42,43], recognized as crucial for effective control of SARS-CoV-2 [44], and leptin reduction may thus imply an impaired immune response. It is important to note that the current study has concentrated on representative adipokines and factors. However, the impact and relevance of other adipokines and molecules should not be dismissed and should be further investigated, especially in the case of factors for which a clear relationship between degree of obesity and abundance is documented (reviewed in [45]).

Lastly, it is essential to address the noticeable enlargement of lipid droplets (LD) within adipocytes. Given the information provided earlier, this enlargement cannot be linked to a more efficient accumulation of triglycerides which on the other hand are impaired. Other potential mechanisms can be considered. For instance, the ability of SARS-CoV-2 to induce cell membrane fusion and syncytia formation is noteworthy, as LDs are encased in a lipid membrane, and fusion among different vacuoles may lead to the creation of larger formations. Spike protein has been implied as the molecular mechanism driving cell membrane fusion: even if the mechanism of syncytia formation during infection is not clearly elucidated, the following mechanism has been postulated [46,47]. During SARS-CoV-2 infection, the protein interacts with the ACE-2 receptor present on the target cell, and subsequently induces fusion of the viral and cellular membranes after undergoing a conformational modification. The presence of Nrp1 and TMPRSS2 proteins on cell surface, in particular, eases the cleavage of Spike protein and increases the syncytia formation. Our findings from SGBS adipocytes transfected with spike cDNA support the notion that the spike protein plays a pivotal role in mediating the fusion of lipid vacuoles. Remarkably, the mere ectopic expression of the gene is potent enough to replicate, albeit to a lesser degree, the LD enlargement effect observed during infection with the complete virus.

Many viruses were reported to interfere with the host cell’s lipid metabolism. Among these, SARS-CoV-2 demonstrates the capacity to influence lipid synthesis and uptake pathways by upregulating the expression of transcription factors associated with lipogenesis and tri-acyl-glycerol synthesis in human monocytes, ultimately leading to the formation of new lipid droplets [48]. Additionally, during viral infection, the core protein of human cytomegalovirus (HCMV) binds to the surface of lipid droplets (LDs) to promote the assembly of viral particles. To sustain elevated levels of constitutive lipid synthesis throughout the infection, HCMV induces the production and enlargement of lipid droplets in human fibroblasts [49,50].

In summary, our findings, leveraging SGBS cells as a valuable tool for investigating SARS-CoV-2’s impact on adipocytes, uncover substantial transformations in this cell type during COVID-19. Notably, we identify an inflammatory surge the fueling systemic inflammation characteristic of the disease, coupled with a diminished endocrine function and triglyceride storage capacity, fostering a state of lipotoxicity and diminished insulin sensitivity. The observed enlargement and fusion of lipid droplets, induced by both active virus infection and Spike protein-only artificial expression, highlight the virus’s ability, likely through its capsid protein, to instigate membrane fusion and generate dysfunctional tissue regions.

## 4. Materials and Methods

### 4.1. Reagents, Plasmids and Antibodies

Chemical and biological reagents were purchased from Sigma-Aldrich/Merck (Milan, Italy), if not-otherwise specified. pcDNA3.1_ spike_del19 was a gift from Raffaele De Francesco (Addgene plasmid # 155297; http://n2t.net/addgene:155297 (accessed on 5 February 2024); RRID:Addgene_155297). The pcDNA3-EGFP was a gift from Doug Golenbock (Addgene plasmid #13031; http://n2t.net/addgene:13031 (accessed on 5 February 2024); RRID:Addgene_13031).

The following antibodies were used throughout this work:

- Anti-NC rabbit polyclonal antibody (GTX135357, GeneTex, Irvine, CA, USA), dilution: 1:1000.
- Anti-GAPDH mouse monoclonal antibody (ab8245, Abcam, Cambridge, UK), dilution: 1:10,000.
- Anti-mouse and anti-rabbit HRP-conjugated antibody (#170-6516 and #170-6515, BioRad, Hercules, CA, USA), dilution: 1:3000.
- Anti-S IgG rabbit monoclonal antibody (40592-V05H, Sino Biological, Beijing, China), dilution: 1:200.
- Anti-N IgG mouse monoclonal antibody (#33717, Cell Signaling, Danvers, MA, USA), dilution: 1:6400.
- Anti-ACE2 IgG rabbit monoclonal antibody (ab15348, Abcam), dilution: 1:200.
- αr488: donkey anti-rabbit monoclonal IgG conjugated to AlexaFluor488 (a21206, Thermo Fisher, Milan, Italy). Immunolabeling dilution: 1/400.

### 4.2. Cell Lines and Virus

Vero E6-TMPRSS2 cells and a clinical isolate of SARS-CoV-2, B.1, were kindly provided by Vita-Salute San Raffaele University Hospital. The manipulation and cell culture propagation of the SARS-CoV-2 isolate was performed at the Virology Unit, Pisa University Hospital, using personal protective equipment and in an approved facility that complied with biosafety level 3 regulations for laboratories, as required for the COVID etiologic agent (https://www.cdc.gov/labs/BMBL.html, accessed on 5 February 2024). Both Vero E6 and Vero E6-TMPRSS2 cells were cultured in DMEM-F12 supplemented with heat-inactivated 10% fetal bovine serum (FBS) (Sigma-Aldrich, Milan, Italy), 2 mM L-glutamine, 100 I.U./mL penicillin, and 100 µg/mL streptomycin (Sigma-Aldrich, Milan, Italy), at 37 °C in the presence of 5% CO_2_. SARS-CoV-2 was propagated in Vero E6-TMPRSS2 cells. Briefly, cells were plated into T75 flasks, and, at about 80% of confluency, were infected with 500 µL of SARS-CoV-2 diluted in 5 mL of medium. Cells were incubated at 37 °C and 5% CO_2_ for 2 h, shaking the flasks every 15 min. At the end of the incubation, the culture medium supplemented with 5% of serum was added, and the cells were incubated until full cytopathic effect was achieved. Cell lysates from the flasks were then centrifuged at 900 g for 10 min and filtered through a 45 µM filter. The supernatants containing the virus particles were aliquoted and stored at −80 °C. The viral titer was calculated from the cytopathic effect (CPE) induced by viral infection in experiments of limited dilution. The viral title was calculated by means of the Spearman–Karber method, and expressed by Median Tissue Culture Infectious Dose tissue/mL (TCID 50/mL). The viral stocks (p3) were titrated both by limited dilution methods and plaque assay. The viral titer calculated by the Reed and Muench method was, respectively, 7 × 10^8^ TCID50/mL.

### 4.3. SGBS Human Preadipocytes Culture and Differentiation

SGBS (Simpson–Golabi–Behemel syndrome) human preadipocytes were cultured in DMEM-Ham’s F12 (1:1) medium containing 2 mM L-glutamine, 100 I.U./mL penicillin and 100 µg/mL streptomycin, 17 uM pantothenic acid, 33 uM biotin, and 10% of fetal bovine serum (FBS). The cells were seeded in 60 mm dishes (Sarstedt, Nümbrecht, Germany) with 5 mL of culture medium, and maintained at 37 °C and 5% CO_2_ until they reached complete confluence. The absence of mycoplasma was checked by a polymerase chain reaction (PCR) analysis (N-GARDE Mycoplasma PCR Reagent set, Euroclone, Milan, Italy, EMK090020). When complete confluence was reached (PID0), white adipogenic differentiation was induced using a serum-free medium supplemented with 100 I.U./mL penicillin and 100 µg/mL streptomycin, 33 µM biotin, 17 µM pantothenic acid, 2 µM rosiglitazone, 10 µg/mL human apo-transferrin, 20 nM human insulin, 25 nM dexamethasone, 500 µM 3-isobutyl-1-methylxantine (IBMX), 100 nM cortisol, and 200 pM triiodothyronine. After four days (PID4), the medium was changed and rosiglitazone, dexamethasone, and IBMX were removed. Cells were maintained in the new medium for the remaining 5 days of differentiation until they reached PID9. The differentiation medium was replaced every third day.

### 4.4. Transfection of SGBS Differentiating Cells

SGBS cells were plated onto a 35 mm glass-bottom dish (#GWSt-3522, WillCo Wells BV, Amsterdam, The Netherlands). At PID6, differentiating cells were transfected with Lipofectamine reagent (Invitrogen), according to the manufacturer’s instructions. After 24 h, the medium was replaced with a 3FC medium until the end of the differentiation process (PID10).

### 4.5. ORO Staining

SGBS cells were seeded in 35 mm glass-bottom dishes (#GWSt-3522, WillCo Wells BV, The Netherlands) and differentiated for 10 days (PID10). Then, the cells were washed in PBS, fixed with 500 µL of 10% formalin in PBS for 1 h, washed in distilled water, and then incubated in isopropanol 60% for 5 min. Isopropanol was then removed and wells were let air-dried for 10 min. Finally, 1 mL of Oil Red O working solution (60% of Oil Red O stock solution in distilled water) was added for 10 min at RT, then removed, and the wells were immediately washed 3 times with distilled water.

### 4.6. Infection of SGBS Cells with SARS-CoV-2

Differentiated SGBS cells plated into a d10 dish were infected with 2 mL of B.1 viral strain (MOI = 1 Vero E6-TMPRSS2); 3 h post-infection, the complete medium was added, and cells were incubated for 48 h. At the end of incubation, the supernatant was recovered, and a second cycle of infection on differentiated SGBS cells was performed. The virus recovered from the second expansion and was used in the following experiments.

For the Western blot and gene expression experiments (digital PCR), differentiated and wild type SGBS cells (2 × 10^4^ cells/well) seeded into 6 well plates, were infected with 600 μL/well of virus. After 48 h post-infection, the supernatants were removed, cells were washed with PBS 1×, and detached by trypsin 1×. Cells were centrifuged at 900× *g* for 10 min and pellets were suspended:

- in Ripa buffer 1× (Millipore 20-188, Burlington, MA, USA) 50 µL/well added with protease inhibitor cocktail 1× (Sigma-Aldrich, P8340) and phosphatase inhibitor cocktail 1× (Sigma-Aldrich, P0044) and stored at −20 °C, for Western blot analysis.
- in QIAzol Lysis Reagent (Qiagen, Hilden, Germany, 79306) 50 µL/well and stored at −80 °C, for gene expression analysis.

For real-time PCR experiments on supernatants, differentiated and wild type SGBS cells (2 × 10^4^ cells/well) seeded into 6-well plates and were infected with 600 μL/well of virus. After 1 h post-infection, the supernatants were removed, cells were washed with PBS 1×, and 2 mL/well of complete medium was added; cells were incubated for 48 h. At the end of incubation, the supernatants were recovered and stored at −80 °C.

### 4.7. qPCR and ddPCR

Virus: The SARS-CoV-2 RNA relative amounts detected for each experimental condition as a cycle threshold (Ct) value were compared, with a mean Ct value determined for the positive infection control. The viral RNA was purified from 100 μL of all cell-free culture supernatants, using the QIAamp Viral RNA Mini Kit (Qiagen, Hilden, Germany). The purified RNA was then used to perform the synthesis of first-strand complementary DNA, using the SuperScript First-Strand Synthesis System for RT-PCR (Thermo Fisher Scientific, Waltham, MA, USA).

Real-time PCR, using the SYBR Green dye-based PCR amplification and detection method, was performed to detect the complementary DNA. We used the SYBR Green PCR Master Mix (Thermo Fisher Scientific, USA), with the forward primer N2F (TTA CAA ACA TTG GCC GCA AA) and the reverse primer N2R (GCG CGA CAT TCC GAA GAA). The PCR conditions were: 95 °C for 2 min, 45 cycles of 95 °C for 20 s, annealing at 55 °C for 20 s, and elongation at 72 °C for 30 s, followed by a final elongation at 72 °C for 10 min. RT-PCR was performed using the ABI-PRISM 7900HT Fast Real Time instrument (Applied Biosystems, Waltham, MA, USA) and optical-grade 96-well plates. Samples were run in duplicate, with a total volume of 20 μL.

Analysis of gene expression: total RNA was extracted from cultured cells using TRIzol (Ambion, Austin, TX, USA). cDNA was synthesized from 0.5 μg of total RNA using iScript Reverse Transcription Supermix for RT-qPCR (Bio-Rad Laboratories, Hercules, CA, USA). PCR mixes for each sample were prepared with cDNA (diluted 1:5) and ddPCR Multiplex Supermix (Bio-Rad Laboratories, USA), loaded in a disposable cartridge (Bio-Rad) together with 70 μL of droplet generation Oil (Bio-Rad), and then droplets were generated by means of QX200 droplet generator (Bio-Rad). In total, 40 μL of droplets were then transferred into a 96-well plate, and an endpoint PCR was performed. Then, the 96-well plate was placed in the QX200 Droplet Reader for detection of positive droplets. The quantification of positive droplets for inflammatory genes (*Hp*, *IL-6*, *mcp1/CCL2*, *PA-I1*), the superoxide-scavenging enzyme *MnSOD*, lipid turn-over genes (*PPARgamma*, ap2/FABP4, *FASN*, *PLIN1*), adipokines (*Adipoq*, *Lep*), and the housekeeping gene *TBP* was performed using the QuantaSoft Software v 1.7 (Bio-Rad). The probes used are the following:

- Hp, IL-6, mcp1/CCL2, ap2/FABP4, Adipoq, Lep, TBP, FAM-conjugated (Applied Biosystems, USA)
- PAI-1, PPARgamma, FASN, PLIN, MnSOD, HEX-conjugated (Bio-Rad Laboratories, USA).

### 4.8. Western Blot

Cells were washed twice with PBS 1X and then were lysed in ice-cold 1× RIPA buffer (Millipore, USA). Lysates were centrifuged at 14,000× *g* for 20 min at 4 °C. Supernatants were resolved by SDS-PAGE (Criterion, Bio-Rad Laboratories, USA). Immunoblots were developed using the Clarity Western ECL Substrate (Bio-Rad Laboratories, USA) and with the ChemiDoc Imaging System (Bio-Rad Laboratories, USA). Densitometric analysis was performed using Image Lab Software v 6.1.0 (Bio-Rad Laboratories, USA).

### 4.9. Immunostaining of Cells 

Adherent cells (untransfected and transfected or infected) in WillCo dishes, after fixing and ORO staining, were rinsed 3 times with 1× PBS and 3 more times with 0.5% Bovine Serum Albumin (BSA) in PBS (PBB). Subsequently, cells were maintained for 40 min at RT in 2% BSA in PBS and rinsed 3 more times with PBB.

For direct immunostaining, SGBS cells (infected, transfected or untreated) were incubated with a primary antibody diluted in PBB, and they were maintained at 4 °C O/N. After rinsing three times with PBB, cells were incubated for one hour with αr488 diluted in PBB. Cells were then extensively rinsed with PBS.

For nuclear staining, immunostained cells were exposed for 5 min to 1 mg/100 mL of Hoechst 33,342 (Thermo Fisher Scientific, USA) in water.

### 4.10. Confocal and ISM Microscopy

Fluorescence was measured by a confocal Zeiss LSM 880 with Airyscan (Carl Zeiss, Oberkochen, Germany) supplied with GaAsP detectors (Gallium:Arsenide:Phosphide, Olympus, Tokyo, Japan). Samples were viewed with a 63× Apochromat NA = 1.4 oil-immersion objective. We adopted a 0.9× zoom for imaging multiple cells (1 pixel = 220 nm) and a 2–6× zoom for imaging single cells; ISM (Airyscan) imaging was carried out at zoom > 3. The pinhole size was set to 44 mm, which corresponds to 1 airy unit (AU) for the green acquisition channel. Pixel dwell time was adjusted to 1.52 µs, and 512 × 512 pixel or 1024 × 1024 images were collected. In confocal mode, we carried out the concomitant acquisition for all channels line-by-line, with the line-average set to 4. In airyscan mode, we carried out a sequential acquisition for all channels with the frame-average set to 4. The acquisition channels were set as follows:

- Blue (Hoechst 33342): λex = 405 λem = 420–500 nm
- Green (Alexa488): λex = 488, λem = 500–560 nm
- Far-red (Oil red): λex = 640, λem = 650–700 nm

The images were visualized and processed by the open-source software Fiji v 2.14.0/1.54f (NIH, Bethesda, MD, USA).

### 4.11. Imaging Analysis of Lipid Droplets

The 16-bit confocal images acquired in the far-red channel relevant to oil-red emission were processed according to a multi-step procedure making use of the in-line available routines of the Fiji software, and are detailed as follows:

A. Gaussian blur of the blue channel image (sigma: 1 pixel).
B. Background subtraction in the blue channel image (rolling ball radius: 25 pixels).
C. Thresholding (Method: Huang).
D. Filling holes and watershed separation of the thresholded image.
E. Particle analysis was performed by setting minimum area size as 4–16 pixels and minimum circularity as 0.05.

This protocol afforded a list of retrieved particles in the image and their area. The area values were subsequently analyzed as frequency distribution and cumulative frequency distribution by Prism 9 (vide infra).

### 4.12. Graphics and Statistics

Graphs were prepared using Prism 9 (GraphPad Software, La Jolla, CA, USA) software. Histogram data are shown as the mean ± SEM, as indicated. Statistical analysis was performed by Prism 9.

## 5. Conclusions

Our findings, leveraging SGBS cells as a valuable tool for investigating SARS-CoV-2’s impact on adipocytes, uncover substantial transformations in this cell type during COVID-19. Notably, we identify an inflammatory surge fueling the systemic inflammation characteristic of the disease, coupled with a diminished endocrine function and triglyceride storage capacity, fostering a state of lipotoxicity and diminished insulin sensitivity. The observed enlargement and fusion of lipid droplets, induced by both active virus infection and Spike protein-only artificial expression, highlight the virus’ ability, likely through its capsid protein, to instigate membrane fusion and generate dysfunctional tissue regions.

These insights carry implications for comprehending the enduring consequences of COVID-19. The adipocyte emerges as a potential reservoir for viral particles, possibly serving as a platform for virus reactivation. Moreover, the alterations documented in this cell type may persist beyond active infection, potentially contributing to the lingering fatigue and chronic inflammation characteristic of the long COVID syndrome.

## Data Availability

Data contained within the article and Supplementary Materials are available on request from the authors.

## Supplementary Materials

The following supporting information can be downloaded at: https://www.mdpi.com/article/10.3390/ijms25042086/s1.
