# Supplementary material for: SARS-CoV-2 Infection Alters the Phenotype and Gene Expression of Adipocytes
Source: Int J Mol Sci. 2024 Feb 8;25(4):2086. doi: 10.3390/ijms25042086 (PMC10889321; doi:10.3390/ijms25042086)
Supplement: Supplementary file 1 [file ijms-25-02086-s001.zip › ijms-2828255-supplementary.pdf]

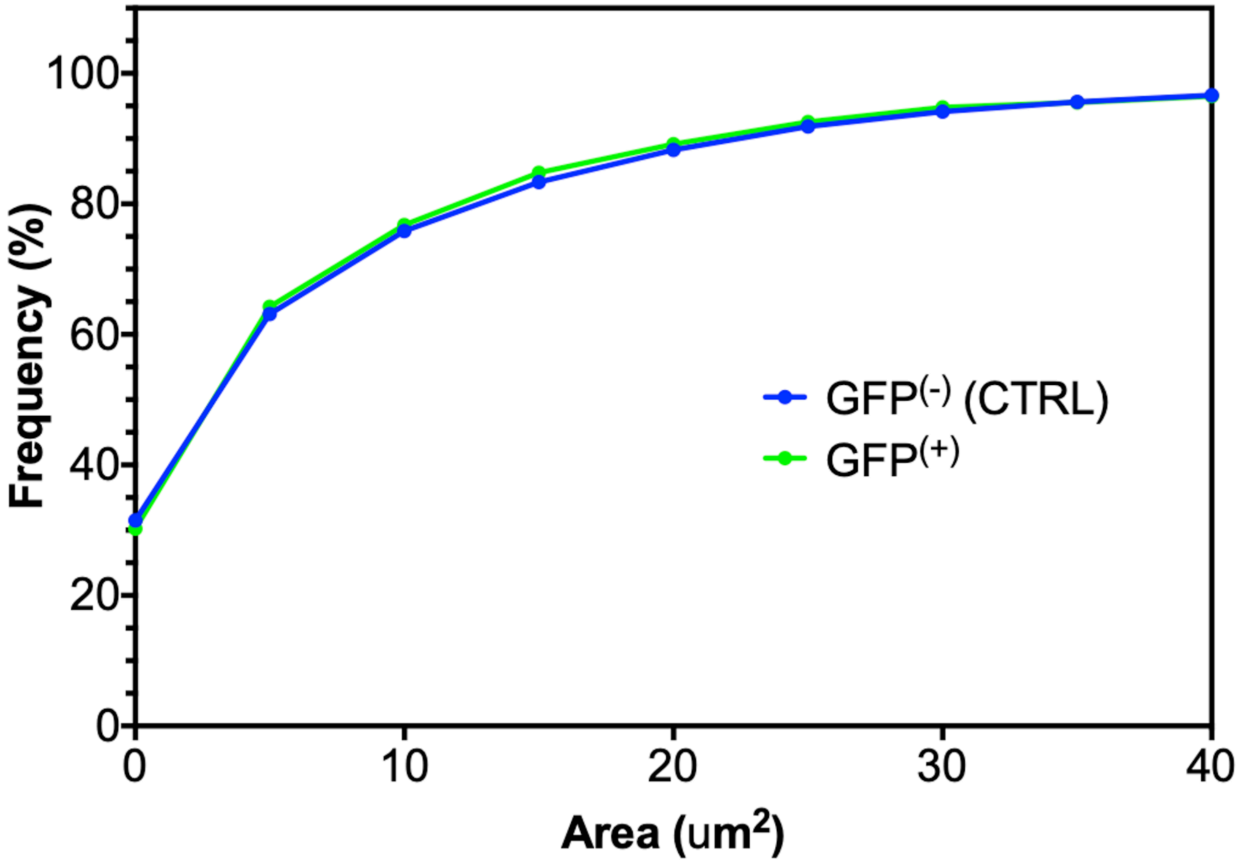

Supplementary Figure S1. Exogenous expression of EGFP protein in SGBS adipocytes does not induce enlargement of LDs as revealed by confocal microscopy

Frequency cumulative distribution of LD areas in in GFP(-) (CTRL) and GFP(+) cells; the cumulative distributions did not resulted statistically different ($p=0.6694$) as assessed by the Kolmogorov-Smirnov test.
